# Exploring the perception and socio-cultural barriers to safer sex negotiation among married women in Northwest Nigeria

**DOI:** 10.1186/s12905-022-01989-3

**Published:** 2022-10-08

**Authors:** Abayomi Folorunso Awoleye, Bola Lukman Solanke, Joseph Ayodeji Kupoluyi, Olufemi Mayowa Adetutu

**Affiliations:** Department of Demography and Social Statistics, Faculty of Social Sciences, Obafemi Awolowo University, Ile-Ife, Nigeria

**Keywords:** Safer sex negotiation, Sexual relationship, Sexual health, Women, Northwest Nigeria

## Abstract

**Background:**

Safer sex negotiation refers to the means through which partners in sexual relationships agree to have intercourse that protects both partners from adverse sexual health outcomes. Evidence is sparse on the socio-cultural barriers to safer sex negotiation, especially in Northwest Nigeria where almost every aspect of women’s lives is influenced by religious and cultural norms. **Understanding the socio-cultural barriers requires having knowledge of the perspectives of community stakeholders such as religious leaders, and community leaders. Thus, from the perspectives of community stakeholders**, this study explored the perception and socio-cultural barriers to safer sex negotiation of married women in Northwest Nigeria.

**Method:**

A qualitative research design was adopted. **Participants were purposively selected across six states, namely, Kano, Katsina, Jigawa, Kebbi, Kaduna, and Zamfara**. **Data were collected through Key Informant Interview (KII). A total of 24 KIIs were conducted using the in-depth interview guide developed for the study. The selection of the participants was stratified between rural and urban areas.** The interviews were tape-recorded, transcribed, and translated from the Hausa language into the English language. Verbal and written informed consent were obtained from participants prior to the interviews. Data were analyzed using inductive thematic content analysis.

**Results:**

**Safer sex negotiation was well-understood by community stakeholders.** Men dominate women in sexual relationships through the suppression of women’s agency to negotiate safer sex. Married women endured domination by males in sexual relationships to sustain conjugal harmony. The practice of complying with traditional, cultural, and religious norms in marital relationships deters women from negotiating safer sex. Other socio-cultural causes of the inability to negotiate safer sex are child marriage, poverty, poor education, and polygyny.

**Conclusion:**

**Community stakeholders have a clear understanding of safer sex negotiation in Northwest Nigeria but this has not translated into a widespread practice of safer sex negotiation by married women due to diverse socio-cultural barriers.** Strategies that will empower women not only to gain more access to relevant sexual and reproductive health information and services but also to encourage women’s assertiveness in family reproductive health decisions are imperative in Northwest Nigeria.

## Background

Safer sex negotiation refers to the means through which partners in sexual relationships agree to have intercourse that protects both partners from adverse sexual health outcomes [1, 2]. Safer sex negotiation is particularly important to promote women’s sexual health in sub-Saharan Africa due to the persistence of patriarchal practices that adversely affect women’s economic and reproductive lives [3–7]. In most cases, these practices manifest in the domination of women by men and in the subjugation of women’s sexual and reproductive health lives to male authority and preferences [5, 8, 9]. Evidence abounds across developing countries that women’s lack of power to negotiate safer sex contributes to poor sexual and reproductive health outcomes, including HIV infection [10–12]. **Safer sex negotiation is thus not only an avenue to enhance women’s sexual and reproductive health outcomes, but also a means to promote gender equity in sexual relationships.** This provides a basis for continued research attention on the underlying factors of poor safer sex negotiation practices of married women in sub-Saharan Africa.

Studies in Nigeria [13, 14, 2] have shown that both married and unmarried women have considerable ability for safer sex negotiation. Many Nigerian women also have positive attitudes toward safer sex negotiation [15]. Nevertheless, they negotiate from a weakened position, often through begging and sometimes disengagement [16]. Other studies outside Nigeria [17–23] have also established that while some women could negotiate safer sex with partners, a substantial proportion of women remain vulnerable to adverse sexual health outcomes due to the inability to negotiate safer sex and lack of capacity to be assertive in sexual relationships [12, 24].

Other studies have identified varied associated factors of safer sex negotiation among women. These factors include high parity [25], the timing of first marriage [2], female genital mutilation [26], community factors [27], improved HIV awareness and perceived empowerment [19], and women’s autonomy [28–30], and intimate partner violence [13]. But these studies are mostly quantitative and less robust in exploring deep-rooted socio-cultural factors that shape safer sex negotiation among married women, especially in a context such as Northern Nigeria. In Northern Nigeria, married women are socially and economically dependent on their partners/husbands and are duty-bound to submit to the dictates of traditional and cultural norms [31]. Though patriarchy is gradually being disrupted by Salafist Islamic practices in Northern Nigeria [32], the region remains largely engendered, and men still dominate women in making sexual and reproductive health decisions.

While the quantitative studies have made important contributions to the knowledge base and improved understanding of the challenges women face in sexual relationships, there is a scarcity of in-depth information about the socio-cultural barriers to safer sex negotiation in Northern Nigeria, where almost every aspect of women’s lives is dominated by religious and cultural practices [33]. Therefore, there is a need to use qualitative research to unravel the deep-rooted socio-cultural issues that hinder safer sex negotiation in the zone. **Understanding these socio-cultural issues requires having knowledge of the perspectives of community stakeholders such as religious leaders, community leaders, and local health care providers, who not only play important roles in shaping community perception of sexual negotiations but also have crucial roles to play in reducing gender inequality within marriages in the community.**

The objective of the study was thus to explore **from the perspectives of community stakeholders**, the perception and socio-cultural factors underlying safer sex negotiation in Northwest Nigeria. **This was done within the context of improving women’s sexual and reproductive health. The study was guided by the research questions: how well do community stakeholders understand safer sex negotiations, and what do community stakeholders perceive to be the socio-cultural barriers to safer sex negotiations among married women?** Findings from the study will provide further inputs for the gender equality and women empowerment concerns of the 2021 revised national policy on population for sustainable development in Nigeria [34]. The policy emphasized gender disparities in every segment of human development in the country and seek to address power relations that deter women from attaining healthy and fulfilled lives.

## Methods

### Study design and settings

The study utilized a qualitative cross-sectional research design. The study was conducted in six states of Northwest Nigeria in 2021. The Northwest Nigeria geo-political zone is one of the zones with the poorest sexual and reproductive health indicators in the country [35]. The average age at first marriage in the zone is 15.8 years, while the average age at first birth is 17.5 years [35, 36]. These two demographic variables contributed largely to the high prevalence of teenage pregnancy and childbearing in the zone. The zone has one of the least levels of modern contraceptive use in the country in addition to having the highest total fertility rate of 6.6 children per woman. Thus, Northwest Nigeria is a high fertility zone [37, 38]. Like in other zones of the country, the proportion of women who are currently married is high in Northwest Nigeria. However, unlike in the southern parts of Nigeria, polygyny is widespread in the zone in line with Islamic practice which is the dominant religion in the zone.

### Participants

Participants were purposively selected across six states, namely, Kano, Katsina, Jigawa, Kebbi, Kaduna, and Zamfara. **Sokoto State was excluded from the study because of recurring terror attacks by the Boko Haram insurgents. We recruited three qualitative researchers from three Universities located within the Northwest zone to identify accessible religious and community leaders in the selected states. The nominated leaders were contacted. While a few declined to participate in the study, others not only consented, but also provided assistance in recruiting other stakeholders such as men leaders, women leaders, and health care providers domiciled in the community. The criteria for inclusion in the study were being currently married, being within the reproductive age of 15–49 for women, being aged 15–59 for men, and being resident in the state where the KII was to be conducted.**

All the participants were adherents of the Islamic faith. The majority of male participants were in the middle age category, while most of the female participants were younger. A large proportion of the participants who were females started childbearing at an age less than 18 years, with most of them having 6 or more children. Six of the participants completed secondary school education, while fourteen attained post-secondary education, and four participants completed only primary education. Most of the respondents were in polygynous unions (Table 1).

**Table 1:** Profile of participants

S/No.	Gender	Age	Type of union	Age at first birth	Children ever born	Education	Religion	State
1	Male	51	Polygyny	22	15	Secondary	Islam	Kano
2	Male	56	Polygyny	23	19	Secondary	Islam	Kano
3	Male	59	Polygyny	28	8	Tertiary	Islam	Kano
4	Female	44	Polygyny	17	5	Secondary	Islam	Kano
5	Female	35	Polygyny	16	6	Primary	Islam,	Jigawa
6	Male	42	Polygyny	22	8	Tertiary	Islam	Jigawa
7	Male	31	Monogamy	26	2	Diploma	Islam	Jigawa
8	Female	28	Monogamy	20	3	Diploma	Islam	Jigawa
9	Female	35	Polygyny	17	4	Secondary	Islam	Katsina
10	Male	42	Polygyny	24	5	Tertiary	Islam	Katsina
11	Male	33	Polygyny	24	5	Tertiary	Islam	Katsina
12	Female	36	Monogamy	16	4	Tertiary	Islam	Katsina
13	Male	60	Polygyny	30	11	Primary	Islam	Kaduna
14	Male	66	Polygyny	26	18	Tertiary	Islam	Kaduna
15	Male	59	Polygyny	27	18	Secondary	Islam	Kaduna
16	Female	46	Polygyny	17	9	Secondary	Islam	Kaduna
17	Male	62	Polygyny	25	14	Tertiary	Islam	Kebbi
18	Female	36	Polygyny	16	6	Diploma	Islam	Kebbi
19	Male	50	Monogamy	30	3	Tertiary	Islam	Kebbi
20	Male	32	Monogamy	27	3	Tertiary	Islam	Kebbi
21	Male	34	Monogamy	17	4	Primary	Islam	Zamfara
22	Male	45	Polygyny	21	8	Primary	Islam	Zamfara
23	Male	57	Polygyny	24	9	Diploma	Islam	Zamfara
24	Female	48	Polygyny	17	6	Diploma	Islam	Zamfara

### Instrument and procedure

The procedure followed to elicit information from community stakeholders such as religious and community leaders, and health care providers domiciled in the communities was the Key Informant Interview (KII). **The KII was suitable for the study because it presents a unique opportunity for capturing the attitudes of religious and community leaders to sexual negotiations, as well as their perspectives on the prevailing nature of sexual behavior in the community. Their opinions on reproductive health issues have been identified to be crucial for the acceptance and promotion of reproductive health services in the region** [39]. **Though it is difficult to prove the validity of their opinions, in most parts of Northwest Nigeria, the opinion of the community and religious leaders are well-respected. Hence, the use of KII in the study was appropriate.**

Four key informant interviews were conducted in each of the aforementioned states. Each of the states was stratified into rural and urban areas. That is, two KIIs were conducted in rural and urban areas, respectively. A total of 24 KIIs were conducted across the study areas between January and March 2021. **An in-depth interview guide was developed for the study.** The interviews aimed to understand participants’ perceptions of safer sex negotiation and the sexual and reproductive health of married women in the community. Participants were asked to describe how married women in the community assert themselves in sexual relationships, particularly how men in the community handle women’s refusal to have sex with partners.

Participants were also asked to narrate how a request for use of a condom if necessary is usually presented to partners. Participants were asked to describe all the factors, particularly the socio-cultural practices that could either facilitate or hinder safe sex negotiation of married women in the community. Participants were also requested to give suggestions on how to improve sexual negotiation between partners in the community. A pilot study was conducted in contiguous areas with similar characteristics to the study areas among some selected participants. The interviews on average lasted between 45 and 60 min. The interviews were conducted in the Hausa language. Six research assistants were recruited and trained for data collection.

### Data management and analysis

The interviews were tape-recorded, transcribed, and translated from the Hausa language into the English language. This was also proofread to confirm accuracy and correctness. An analytical framework and codes were developed. Coding was used to break down the transcribed data into units of meaning which were categorized, labeled, and subsequently organized into themes and sub-themes. Following the refinements, linkage, and integration of categories around a core, the emerging constructs were used to gain insights on barriers to safer sex negotiation-related issues among the participants. A note taker was present for observations, note-taking, and the use of a tape recorder to recode the conversation. The audio files were extracted from the tape recorder, using universal serial bus cables for transferring the file to a laptop for proper labeling, identification, and documentation. Data were analyzed using inductive and thematic content analysis. ATLAS. ti8 software was used for coding the transcripts.

## Results

### Perception and practice of safer sex negotiation

Safer sex negotiation was widely perceived to be a couple’s affair that should be discussed covertly but clearly understood to mean communication between husband and wife about how to safeguard family life through sexual intercourse that reduces the risk of sexually transmitted infections and unintended pregnancies. It was also perceived as a means to enhance optimal child spacing and sexual satisfaction. However, practicing safer sex negotiation in unions was believed to be an adverse consequence of exposure to western civilization which was believed by participants to be associated with increased female education, empowerment, and monogamous marriage. This was evident in a participant’s response who opined that:

*“The causes are education, westernization, civilization, high income, being alone or being the only wife, imbibing other cultures and values and employment and women whose husbands are irresponsible (i.e., they do not take care of their wives)” (*Participant, Kaduna State)

Sexual negotiation was considered unacceptable, taboo, and lacked respect for the male partner. Most participants held the view that women in the study areas cannot negotiate for safer sex because men are considered to be supreme and women must obey the views of their partners. Two participants opined that:

Our practice here is that we believe that a woman does not have a say or to approach her husband on her sexual wishes. The woman must obey

(Participant, Kaduna State)

Unless there is lack of trust between the wife and the husband, but in the absence of that, such negotiations for safer sex are not allowed

(Participant, Kebbi State)

Virtually all the female participants expressed little support for safer sex negotiation due to the serious consequences that may arise from the attempt to negotiate. Consequences as reported by participants include spousal violence and marital disharmony, and sometimes marital dissolution. Some participants, however, reported that safer sex negotiation may be practiced if it is evident that the male partner is not responsible for the wife or children’s upkeep.

### Socio-cultural barriers to safer sex negotiation

#### Child marriage

Participants reported that child marriage which is a common practice in the study location hinders most women from safer sex negotiation because most of the girls are considered to be too young at the time of marriage. Participants observed that wide age differences between partners in marital relationships in the community worsen the hitherto subordinate position of women in society. It was narrated that early entrance into marital unions truncates young women’s ability to exert their agency to demand safer sex even when it is well known to them that their husbands have multiple sexual partners. In addition, participants reported that counseling and advice provided to young girls during marriage ceremonies in the community implore young married girls to be obedient to partners’ directives even when detrimental to their health and well-being. One participant narrated that even if the male partner desire to explore various sex acts, the duty of the young married partner is to comply. Another participant further reported that sexual negotiation was inconceivable to married young women even when they are being coerced to have sexual intercourse. Examples of the hapless situation of some married young girls were provided by participants. According to three participants:

*“Traditionally in this community, when a girl gets married and is taken to her husband’s house, one of the advice given to her is that she should always obey her husband no matter what’’* (Participant, Zamfara State)

*“Some might use other traditional or modern methods, such as putting the drugs in his wife’s tea to drink or inside food for her to fall asleep, so that he can have sexual relations with her”* (Participant, Zamfara State)

*“Yes, we have such problems here. Those scenarios can certainly be found here. There are situations, whereby a woman has done what she thinks is right but the husband is not satisfied and he will try to forcefully do it the way he wants”* (Participant, Zamfara State)

#### Poverty

It was reported that women must obey their husbands whenever they wanted to have sexual intercourse because the husbands are the breadwinner and as such, they wield enormous power in household decision-making. Not resisting unsafe sex is influenced to a larger extent by economic insecurity and heightened financial vulnerability of women. Satisfying men sexually in the context of unprotected sexual intercourse has been a coping strategy to stave-off household malnutrition and a life of privation. It was reported that most women depend on their husbands for survival and as a result women might not have the right to negotiate for safer sex. What could be gleaned from the participants is that women’s total reliance on male partners for economic and financial support disempowers them from exerting their agency in negotiating for safer sex. An extract from an interview reads thus:

*“Some of the factors include dependency on the husband, the husband is the bread winner, and the wife’s lack of authority over marital issues.* (Participant, Kano State)

#### Male dominance and attitude

Participants stated that their culture supports the dominance of men in household decision-making. Culturally, husbands’ decision in the family is supreme. Men decide when sexual intercourse takes place and all other sexual activities. In addition, it was found that it is culturally unacceptable for a wife to deny her husband sexual intercourse because the culture respects and values large family size. Though, few of the respondents indicated that culture has been replaced by religion. It was noted that their religion shares similar characteristics with the indigenous culture. According to some of the participants, safer sex negotiation is not totally acceptable based on the tenets of the Islamic religion.

Except on the grounds of poor health, women cannot deny their husbands the right to have sex with them. However, according to one of the community leaders, religion allows for negotiations on the number of children and the use of family planning but it was reported that this is rare in practice. By and large, most of the participants lay emphasis on the husband’s supremacy as a strong factor that influences women’s ability on safer sex negotiations even in the context of the Islamic religion. Some of the participants opined that:

*The cultural norm, therefore, is the fact that our culture gives supremacy to the husband. The wife must obey whatever the husbands want.*

(Participant, Kano State)

*I cannot remember but our culture does not give women an outright right to make negotiations on sexual and reproductive rights.*

(Participant, Kano State)

*The challenges women faced included religious bounds. According to religion, women are obliged to respect and surrender to the sexual urge of their husbands* (Participant, Kano State)

*“If you come here, you will see that most of us are Muslims and basically Hausa- Fulani. You will see that the religion does give women equal rights to actually negotiate this kind of thing, and ah so is a bit of misunderstanding, especially among us here as some think it’s actually the religion that gives the men the absolute power which is actually not true basically”.*

(Participant, Katsina State)

Most of the participants described males’ attitudes towards safer sex negotiation as unfavorable and non-supportive. Many undesirable words and phrases were used to describe men’s attitudes to practices of safer sex negotiation. For example, some participants were of the view that men are ‘arrogant’ or ‘dictators’ while some others were of the view that men enjoy using ‘power’ to have their way in sexual relationships. Notwithstanding, a participant reported that some women in the community do resist being dominated by men on sexual matters.

*As a result of this, the man can hurt his wife’s private parts and she can get injured and become sick as a result.*

(Participant, Katsina State)

*Some men are not easy to reason and understand the needs of women.*

(Participant, Kano State)

#### Women’s education

Participants narrated how education plays a vital role in women’s economic and reproductive lives in the community. Participants observed that with improved education, women have the privilege of information and education about how to live healthy and productive lives. It was stated that education also attracts respect and dignity to women. Most of the participants however lamented that formal education is not widespread among women in the zone. It was also reported that some young women stopped schooling as a result of early marriage and childbearing. According to participants, most of the women found it very difficult to continue their education, which often limits their economic value in society. One of the participants stated that women who are well educated would be able to negotiate safer sex, while the practice of safer sex negotiation is inconceivable to poorly educated women:

*Second, lack of education, many women do not know about their rights or the importance of health in their marital lives. Many also do not know the right of women concerning marriages or the importance of abiding by healthy sexual practices.*

(Participant, Kano State)

*The problem is that we have a lot of married women that are actually struggling to have a say or have knowledge about sexual and reproductive issues.*

(Participant, Katsina State)

*Yes, in such a way that most of them are not allowed to further their education. So, they are not able to know much about sex or how marital issues are resolved especially concerning sex and how it can be very open between husband and wife* (Participant, Katsina State)

#### Spousal violence and male controlling behavior

It was reported that many women in the zone are scared of the serious consequences of safer sex negotiation. It was stated by participants that some women experienced spousal violence and marital disharmony when they attempt to negotiate safer sex with partners. Some of the consequences stated include fear of divorce, social stigma, marital dissolution, and sexual abuse or marital rape. The women thus tolerate unsafe sexual practices because their husbands often misunderstand such discussions. Some of the participants also reported that unsuccessful safer sex negotiations may fuel discontent among couples because the male partner may either think that the woman is no longer worthy of trust or become uncontrollable.

*Secondly, the fear of divorce. Since it is culturally acknowledged that it is men that seek women’s hand in marriage, trying to negotiate may evoke the anger of the husband and may lead to divorce*

(Participant, Kano State)

*Yes, there are challenges, just as I said earlier on, a woman may lose her marriage if she dares make attempt to seek her rights as a wife, and looking at the number of single ladies, widows, and those divorced, most of the married women are not ready to have their matrimonial homes broken for whatever reason*

(Participant, Jigawa State)

#### Family and communal support

The participants clearly stated that inadequate family and community support hinder women’s ability to negotiate safer sex. As earlier observed, safer sex negotiation results in marital disharmony, which might require the support of family or community members to resolve. But the experience of most of the participants was that the women are not likely to get such support if the cause of the conflict is sexual intercourse between husband and wife. It is expected that the woman was married to play some roles in the marriage which include being available at any time the partner desire. A participant opined that:

*Honestly, there are cultural constraints peculiar to us, take, for example, an instance where a married woman takes her complaints to her parents or other family members for redress, in most cases you find that her family will not properly entertain such complaints, which make it very difficult for the woman to overcome the problem. The women are often persuaded to go back and be patient because the cultural practice is to tell them that marriage is all about being patient.*

(Participant, Jigawa State)

#### Polygyny

Most women in the study areas are in polygynous unions. Participants reported that sexual negotiation is difficult due to competition for love, care, and household resources from the husband. One of the participants stated that women are bound to lose the love and respect of their husbands if they negotiate for safer sex because there are other wives in the household who are ready to meet the desire of the man. One participant stated that sexual negotiation is only feasible if the man is not responsible for family welfare. According to the participant:

*If a woman in a polygamous home tries to express her wishes or negotiate sex she may lose the love and respect of her husband which may even lead to dispute or dissolution of marriage.*

(Participant, Kano State)

*For women in a polygamous home, negotiation is taken to mean a lack of care to the husband.*

(Participant, Kaduna State)

## Discussion

This study explored perception and socio-cultural issues that hinder safer sex negotiation practice in Northwest Nigeria. It builds on existing knowledge that married women in Nigeria have the ability to negotiate safer sex with partners [13, 14, 2, 15], but extended the frontiers of knowledge by undertaking a robust exploration of deep-rooted socio-cultural practices that shape married women’s potential to negotiate safer sex in Northwest Nigeria, where most married women are not only socially and economically dependent on their male partners, but are also duty bound to submit to the dictates of religious and cultural norms [31] even with the rising profile of Salafist Islamic practices in the zone [32]. The study also provides additional information beyond the findings in numerous quantitative studies [2, 13, 14, 25–30]. It was evident in the study that safer sex negotiation as well as its importance for women’s sexual and reproductive health was well-understood by women in Northwest Nigeria. Married women in the zone, however, face diverse socio-cultural challenges to transform their knowledge into sexual and reproductive behaviors. Thus, improving women’s sexual and reproductive health in Northwest Nigeria requires strategies that will empower women not only to gain more access to relevant sexual and reproductive health information and services but also to encourage women’s assertiveness in family reproductive health decisions. As rightly observed in the 2021 revised population policy [34], existing power relations within most Nigerian households undermine women’s attainment of healthy reproductive and economic lives. This unequal power relation could be addressed frontally through more community sensitization and the enactment of laws focusing on discriminatory and harmful practices against women within the family in Northwest Nigeria. Four other key findings emerged from the study.

One, early marriage was found to be a practice that undermines safer sex negotiation. Our study revealed that child marriage promotes subordination of women in making household decisions and thus makes women tolerate unsafe sexual practices of their partners to sustain marital harmony. This finding is in consonance with earlier studies [2, 11]. The plausible explanation for this observation is attributable to the fact that child marriage is one of the manifestations of gender-based inequality as women who married very early lack the autonomy to exert their agency in negotiating safer sex. The wide age difference between partners makes women subservient in sexual and reproductive health decisions because early marriage mirrors disadvantaged socio-economic conditions. The policy implication of this finding is that younger women are at heightened risk of unsafe sexual practices because of the inability to exert individual agency to resist unsafe sexual practices from their husbands. There is a need for more empowerment programs that delay marriage or promote safer sex negotiation in marriage. In addition, the governments of Northwest zones, need to urgently encourage the enactment of legislation to raise the minimum age at first marriage to eighteen years in the zone. But this should be preceded by appropriate sensitization of religious and community leaders in the zone.

Two, the study reveals that poverty among women was a barrier to safer sex negotiation practices in the study area. A similar finding was observed in a number of existing studies conducted across sub-Saharan Africa [11, 27, 29, 30]. A plausible explanation for this finding is attributed to the financial vulnerability of women and absolute reliance on husbands for household expenditures. There is a need to improve the economic empowerment of Northwest Nigerian women. This could be achieved in at least two different ways. One, the provision of skill acquisitions will not only provide the opportunity of earning independent income for some of the women, but it will also boost their self-esteem and thereby enhance their assertiveness in marital relationships. Two, sexuality education programs targeting poorly educated women could be developed for implementation in the zone. The program should target more out-of-school women who may now be dominant in Northwest Nigeria due to the persistence of armed insurgency and banditry in the zone. However, such a program should be sensitive to the culture and traditions of people in the zone to boost its acceptability.

Three, this study found that women’s educational attainment shapes their ability to negotiate safer sex in agreement with findings in previous studies [2, 14, 15, 29, 30]. The likely explanation for this is that education enlightens and influences women’s ability to resist cultural practices that promote unsafe sexual practices and their negative effects. Virtually in all population and health programs in Nigeria, education continues to attract important policy attention. However, in Northwest Nigeria, education particularly women’s education remains low in spite of the implementation of a universal basic education program in the country. This may have denied many women in the zone the opportunity of acquiring formal population and family life education which is included in the primary and secondary school curricula in the country. This establishes the need for special sexuality and family life education programs to target uneducated and poorly educated women in the Northwest zone. Such a program is likely to boost more acceptance and practice of safe sexual behavior among young girls and married women in the study area.

Four, it was revealed in the study that male dominance and attitude adversely affect safer sex negotiation. It was dominant in the narratives of the participants that women do not have much say in their sexual relationships and that only men have the power to decide on sexual matters, including the nature of sexual intercourse and other family planning issues. This finding aligns with previous studies that explained that men wield enormous power regarding decisions as to whether sexual intercourse should be protected either to prevent pregnancy or sexually transmitted infections and that women are at the mercy of their husbands in negotiating safe sexual practices [11, 18, 40]. Furthermore, this current study showed that the practice of religion and culture played a significant role in determining the extent of safer sex negotiation because most women were guided by their cultural and religious beliefs in their various communities despite their level of education. This finding corroborates the position of earlier studies [11, 39], and justifies the need for a behavior change program that dispels deep-rooted socio-cultural and religious practices that adversely affect the health and sexual well-being of women. Such programs should pay special attention to women in polygynous unions. As revealed in the study, polygyny does not promote safer sex negotiation. This finding substantiates findings in earlier studies where it was found that women in polygynous unions had lower odds of safer sex negotiation [2, 14, 20]. It is a known fact that the availability of other wives in the household reduces women’s sexual assertiveness. This however does not imply that women in polygynous unions should not be protected from harmful or unprotected intercourse. It is thus necessary for the behavior change program to devise means of incorporating the particular concerns of women in polygynous unions.

### Strengths and limitations

This study has been able to critically explore socio-cultural factors that militate against safer sex negotiation through a qualitative research method. **The study has a major strength in that it explored issues that could not be teased out in most quantitative studies**. **By shedding light on how community stakeholders perceived safer sex negotiation, the study reveals key concerns that could be integrated into sexual and reproductive health initiatives in Northwest Nigeria.** However, one of the limitations of this study is that the study did not interview couples. **Attempts to conduct focus group discussions where couples could share lived experiences were made impossible due to rising cases of armed insurgency and banditry in the Northwest zone. The lack of couples’ perspectives might have implications for some of the results of the study.** This is because safer sex negotiation is expected to take into cognizance the views of couples who had lived the experience so as to derive robust and in-depth findings from the study. In addition, the study cannot be generalized to the whole country because it focused on a few participants from Northwest Nigeria. However, this study helps to identify socio-cultural factors influencing safer sex negotiation in the study area.

## Conclusion

Safer sex negotiation was well-understood by community stakeholders. The major socio-cultural barriers to safer sex negotiations among married women in Northwest Nigeria include child marriage, male dominance, poverty, spousal violence, male controlling behavior, poor women’s education, and lack of family and communal support. This study showed convergent discourses on some deep-rooted socio-cultural and gender norms that influenced safer sex negotiation. It revealed a nuanced position on male dominance in marital sexual relationships, child marriage, poverty, polygyny, and women’s educational status. Governments in Northwest Nigeria need to devise strategies that will empower women to gain more access to relevant sexual and reproductive health information, as well as initiatives to encourage women’s assertiveness in family reproductive decisions.

## Data Availability

The datasets analysed in the study are available from the first author on reasonable request via. awoleyeabayomi@gmail.com.

## Declarations

### Authors’ information

AFA is a doctoral graduate of Demography and Social Statistics, Obafemi Awolowo University, Ile-Ife, Nigeria, while BLS, JAK, and MOA are Faculty Members in the Department.

## List of abbreviations

KII: Key Informant Interview.
NPC: National Population Commission.
